# Molecular Modification of Kex2 P1’ Site Enhances Expression and Druggability of Fungal Defensin

**DOI:** 10.3390/antibiotics12040786

**Published:** 2023-04-20

**Authors:** Yanjie Jin, Na Yang, Da Teng, Ya Hao, Ruoyu Mao, Jianhua Wang

**Affiliations:** 1 Gene Engineering Laboratory, Feed Research Institute, Chinese Academy of Agricultural Sciences, Beijing 100081, China; 2 Innovative Team of Antimicrobial Peptides and Alternatives to Antibiotics, Feed Research Institute, Chinese Academy of Agricultural Sciences, Beijing 100081, China; 3 Key Laboratory of Feed Biotechnology, Ministry of Agriculture and Rural Affairs, Beijing 100081, China

**Keywords:** fungal defensin, *Pichia pastoris*, P1’ site of Kex2, expression, druggability

## Abstract

*Pichia pastoris* is the widely used expression system for producing recombinant secretory proteins. It is known that Kex2 protease plays a vital role in the process of protein secretion, in which the P1’ site affects its cleavage efficiency. To enhance the expression level of fungal defensin-derived peptide NZ2114, this work attempts to optimize the P1’ site of Kex2 by replacing it with 20 amino acids in turn. The results showed that when the amino acid of the P1’ site was changed to Phe (F), the yield of target peptide significantly increased from 2.39 g/L to 4.81 g/L. Additionally, the novel peptide F-NZ2114 (short for FNZ) showed strong antimicrobial activity against Gram-positive (G^+^) bacteria, especially for *Staphylococcus aureus* and *Streptococcus agalactiae* (MIC: 4–8 μg/mL). The FNZ was very stable and retained high activity in various conditions; in addition, a low cytotoxicity and no hemolysis were observed even at a high concentration of 128 μg/mL, and a longer postantibiotic effect was reached. The above results indicate that this engineering strategy provided a feasible optimization scheme for enhancing the expression level and druggability of this antimicrobial peptide from fungal defensin and other similar targets by this updated recombinant yeast.

## 1. Introduction

It is well known that *Staphylococcus aureus* (*S. aureus*) is one of the most common Gram-positive (G^+^) pathogens. It leads to various human and animal diseases, such as skin infections, pneumonia, pyemia, infective endocarditis, and other serious diseases [1]. For example, *S. aureus* can cause direct infection with mastitis in animals [2], when it co-infects with avian reovirus and then leads to severe avian arthritis [3]. What is worse is that *S. aureus* is very difficult to completely eliminate by traditional antibiotics due to its high resistance [4]. The antibiotic resistance is mainly from the overuse and leads to the perceived screening of bacteria. More antibiotics entering into animals induces formation of antibiotic-resistant bacteria, and their excretion through feces will result in an obvious increase in the abundance of antibiotic-resistant bacteria. Antibiotic-resistant bacteria also destroy the stability of intestinal flora and affect the body’s digestive and immune system [5,6,7]. For decades, vancomycin, a glycopeptide antibiotic, has been one of the first-line treatments for MRSA infections [8]. However, with the increase of its clinical application, the first case of vancomycin-resistant *S. aureus* appeared in Japan in 1997, and then similar cases were reported in other countries [9,10,11]. In 2017, vancomycin-resistant MRSA was classified as a high-risk drug-resistant bacterium by WHO [12]. Hence, there is an urgent need to find new antibacterials with potent bactericidal activity and low drug resistance.

Antimicrobial peptides (AMPs) are a group of short peptides widely distributed in almost all forms of life [13]. Due to the high sensitivity and low resistance properties, AMPs have become one of the potential novel drug candidates during the 21st century; our team has recently proposed the iron triangle theory of health protection from AMPs, antibiotics, and vaccines and a frame of AMPs development for the post-antibiotic era [14,15]. In a special scope, AMPs show a potential to replace traditional antibiotics; but in a higher and broader scope, they actually belong to one of the new types of antimicrobials. Plectasin is derived from the saprophytic ascus fungus (*Pseudoplectania nigrella*) as the first AMP fungal defensin with a typical CSαβ structure [16] and with a good bactericidal effect against G^+^ bacteria by binding with cell wall component lipid II [17]. There is a good preliminary foundation in our team regarding the modification, expression, and efficacy studies of this class of antimicrobial peptides, which are expected to have good development prospects for antibiotic substitution and combination administration to hinder drug resistance [18,19,20,21,22,23]. We know that defensins take over half of AMPs in quantity from the AMP databases [24,25]. Looking back and thinking about the historic experience during the discovery of new antibiotics, we are impressed by the fact that, on one hand, the most defensin produced by fungi or other microorganisms has the better druggability and is more suitable being as new sources of candidate antimicrobials to dig and follow, which is superior to that of plants and animals with more difficulties to validate their druggability. On the other hand, the high cost and lower stability have always been the first key factors limiting the clinical application of AMPs [17,26], which is the basic premise and panorama that all AMP colleagues devoted to clinical application must face squarely or directly. Therefore, it is of great significance to modify the production process of microbial AMPs including the heterologous expression and biological preparation of defensin. *Pichia pastoris* (*P. pastoris*) has been successfully used in industrial and medical fields due to its merits such as post-translative modification, easy genetic manipulation, no toxin, and stability of recombinants [27]. The NZ2114, a mutant of plectasin with the sequence of 40 amino acids as GFGCNGPWNEDDLRCHNHCKSIKGYKGGYCAKGGFVCKCY (measured average molecular mass 4410.6 Da), was successfully expressed via the *P. pastoris* system in our laboratory at a level of 2390 mg/L (ferment supernatant) [18]. The rNZ2114 maintained its original structure as a CSαβ with three disulfide bonds (PDB No. 6K50) and showed strong bactericidal activity against drug-resistant G^+^ bacteria, especially for *S. aureus* [28].

In fact, non−specific cleavage of plectasin and its derivates always exists during our previous works [18,20,29], there is a larger room for improvement on higher specific cleavage efficiency. The cleavage efficiency of signal peptide is an important factor affecting protein expression in *P. pastoris*. Kex2 is a Ca^2+^-dependent serine protease derived from *Saccharomyces cerevisiae* [30]. It can specifically recognize and cut the carboxy–terminal peptide bond of Arg–Arg and Lys–Arg amino acids. In yeast, Kex2 is mainly responsible for cutting the signal peptide in the precursor sequence, releasing the mature heterologous protein [31]. Previous studies have reported that the cleavage ability of Kex2 is positively correlated with the secretion of exogenous proteins from yeast. Optimization of the cleavage site by Kex2 effectively improved the secretion efficiency [32]. It was shown that Kex2 family enzymes can interact with the P1’, P2’, P3’, and P4’ side chains at substrates [33], in which the binding with P1’ is more specific and stricter [34]. It was reported that the modification of the P1’ site significantly improves the production of exogenous secreted proteins [35]. However, a low preference of selectivity for most residues at the P1’ site by Kex2 depends on the residue at the P3’ position and its binding to the S3 subsite [36]. In this study, NZ2114 was chosen as the object peptide, and the P1’ site amino acid with optimal cutting efficiency was screened; both expression and druggability of the candidate F-NZ2114 (short for FNZ) of the AMP derivative were further studied.

## 2. Results

### 2.1. Construction of Recombinant Plasmids

**Figure 1:**
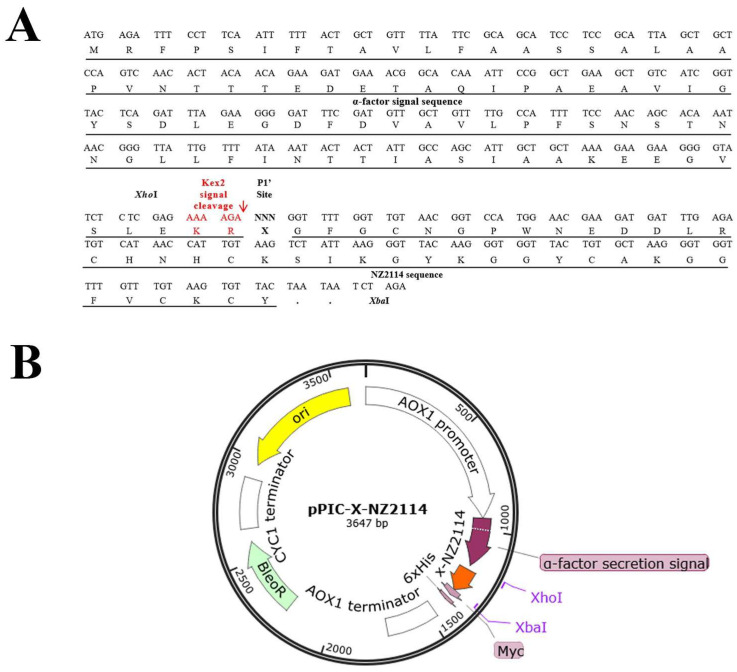
The construction of the pPIC-X-NZ2114 plasmid. (**A**) The amino acid sequence and corresponding nucleotide sequence of α-signal peptide and NZ2114. The α-factor signal is the α-MAT factor signal peptide from *Saccharomyces cerevisiae*. The X indicates 20 amino acids at the P1’ site, “NNN” represents the three bases that code for “X” in the P1’ site. The Kex2 enzyme signal recognition site is marked in red and the arrow represents the Kex2 signal cleavage site. (**B**) Schematic representation of the recombinant expression plasmid pPIC-X-NZ2114.

The basic expression cassette consisted of the sequence of *Xho*I, Kex2, P1’ site, NZ2114, stop codon, and *Xba*I. Twenty sequences were designed, each with a different P1’ site. As shown in Figure 1, the codon-optimized DNA sequences were digested with *Xho*I and *Xba*I and connected to the original yeast secretory expression vector pPICZαA. The amino acid sequences of X-NZ2114 (short for XNZ) are listed in Table S1. The positive colonies were initially screened and subjected to colony PCR confirmation after transforming into the *P. pastoris* X-33.

### 2.2. Selection of Positive Transformants at the Well Plate, Shake Flask Level

**Figure 2:**
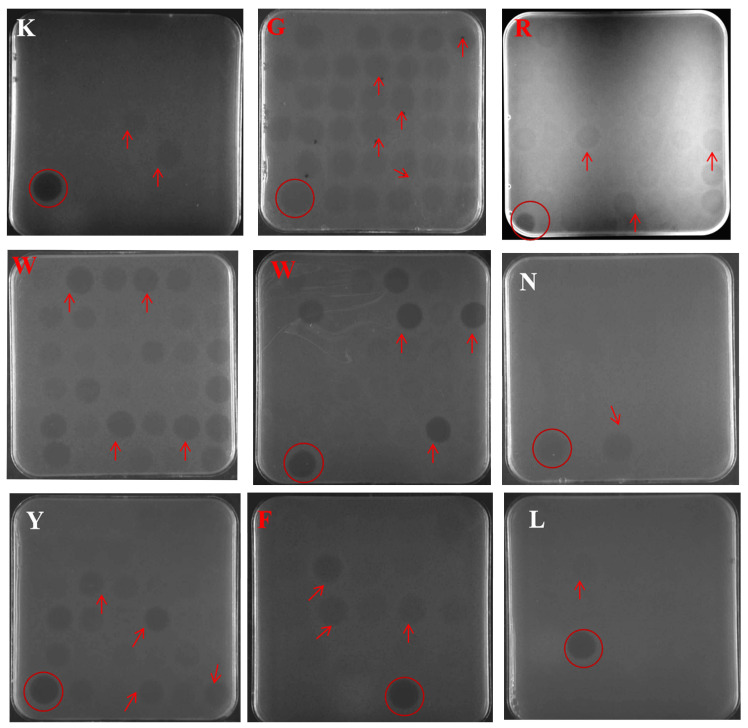
The inhibition zones of XNZ fermentation supernatants in 48-well plates. The circle is marked as the NZ2114 control; the arrow represents the screened positive transformants.

**Figure 3:**
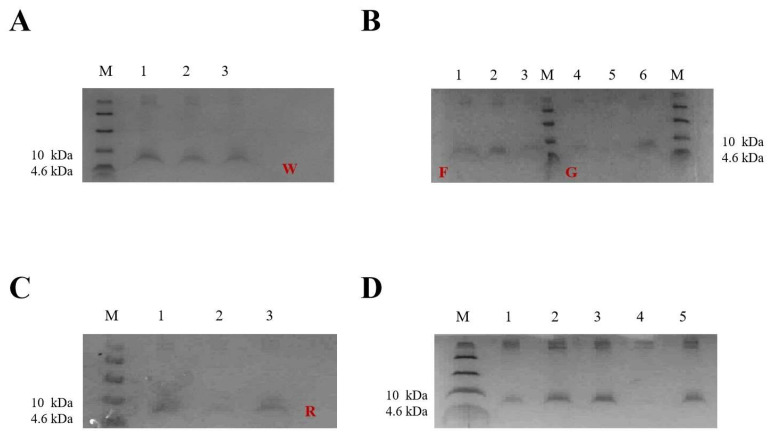
Tricine SDS–PAGE analysis of XNZ fermentation supernatants in 250 mL and 1 L shake flask levels. (**A**) Transformants of WNZ in 250 mL flask level. M, low-range protein ladder; Lanes 1 and 2, transformants of W25, W28; Lane 3, transformant of NZ2114, respectively. (**B**) Transformants of F/GNZ in 250 mL flask level. M, low-range protein ladder; Lanes 1 and 2, transformants of F34 and F39; Lanes 5 and 6, transformants of G1 and G12; Lanes 3 and 4, transformant of NZ2114. (**C**) Transformants of RNZ in 250 mL flask level. M, low-range protein ladder; Lanes 1 and 2, transformants of R27 and R33; Lane 3, transformant of NZ2114. (**D**) Transformants of W/R/G/FNZ in 1 L flask level. M, low-range protein ladder; Lane 1, transformant of NZ2114. Lanes 2 to 5 are the transformants W25, R33, G1, and F39, respectively.

As shown in Figure 2, positive transformants (96 for each sequence) were selected from YPDS plates containing zeocin resistance and subsequently induced in 48-well plates for 120 h with methanol as the inducer. The fermentation supernatant of colonies with the P1’ site of Lys (K), Gly (G), Trp (W), Arg (R), Asn (N), Tyr (Y), Phe (F), and Leu (L) have better antibacterial activity (Figure 2). According to the induction protein results of 250 mL shake flask level by electrophoresis gel (Figure 3A–C), four colonies of W25, R33, G1, and F39 were further selected for induction at 1 L shake flask level (Figure 3D) with the yields of 0.33, 0.23, 0.09, and 0.27 g/L, respectively.

### 2.3. Expression of W25, R33, G1, and F39 in Fermenter Level

**Figure 4:**
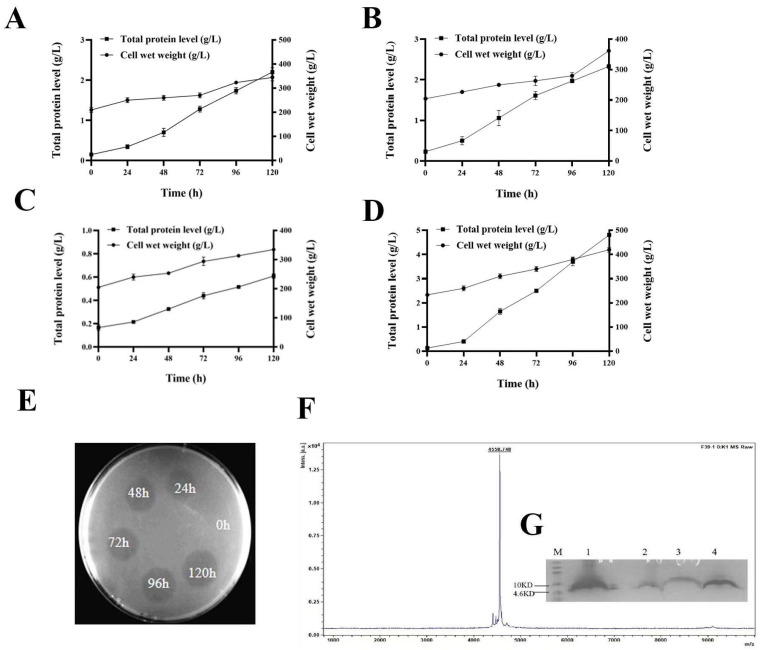
Expression of XNZ transformants in 5 L fermenters and purification. (**A**–**D**) Time curves of the total secreted protein levels and cell wet weights of transformants W25, R33, G1, and F39, respectively; (**E**) The inhibition zones of FNZ fermentation supernatants with different induced time against *S. aureus* ATCC43300; (**F**) MALDI–TOF MS analysis of the purified FNZ; (**G**) Tricine–SDS–PAGE analysis of the cation-exchange chromatography purification of FNZ. Lane 1: 120 h fermentation supernatant; Lane 2: penetration peak; Lane 3: 27.5% B-eluting peak; Lane 4: 60% B-eluting target peak.

The recombinants W25, R33, G1, and F39 were subjected to high−density fermentation in 5 L fermenters. The cell wet weight increased with induction time and up to 354, 362, 330, and 420 g/L at 120 h of induction. The total protein levels were 2.26, 2.33, 0.60, and 4.81 g/L (supernatant), respectively (Figure 4A–D). The specific production rates were 0.053, 0.057, 0.015, and 0.095 mg/g/h for W25, R33, G1, and F39, respectively. Additionally, the antimicrobial activity was enhanced with the increase of the expression level accordingly (Figure 4E). Due to the extremely high yield, recombinant F39 and its product FNZ were selected for the next study.

### 2.4. Model Structures of Wild-Type and Mutant Pro-Signal Peptides

**Figure 5:**
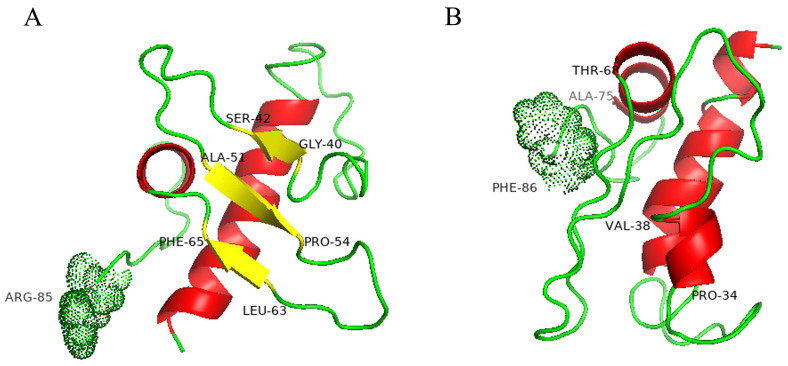
The 3D simulation structures of original α-MAT signal peptide (**A**) and P1’-modified α-MAT signal peptide (**B**).

The I-TASSER analysis was used to structure simulation of the pro-signal peptides of wild-type and FNZ (Figure 5). By comparison (Figure 5A,B), the sequences of amino acid 40–42, 51–54, and 63–65 in the wild-type α-mating factor (α-MAT) formed a β-fold, but sequences of amino acid 34–38 and 68–75 in FNZ formed an α-helical structure. This change in the secondary structure of the pro−region was considered to prevent probably the aggregation of target peptides; the exact mechanism should be a prioritized study in future.

### 2.5. Purification of FNZ

As shown in Figure 4G, the FNZ was purified and a single target band (Lane 4) was detected, which displayed potent antimicrobial activity (Figure S1). The average molecular mass of recombinant FNZ measured by MALDI–TOF MS was 4558.748 Da, which was consistent with the theoretical molecular weight. Nevertheless, there was a 6 Da less than the molecular value of 4564.2 Da predicted by ExPASy software, because the six hydrogen atoms lost from the forming of three pairs of disulfide bonds have not been included and calculated.

### 2.6. Secondary Structure Determination of FNZ

**Figure 6:**
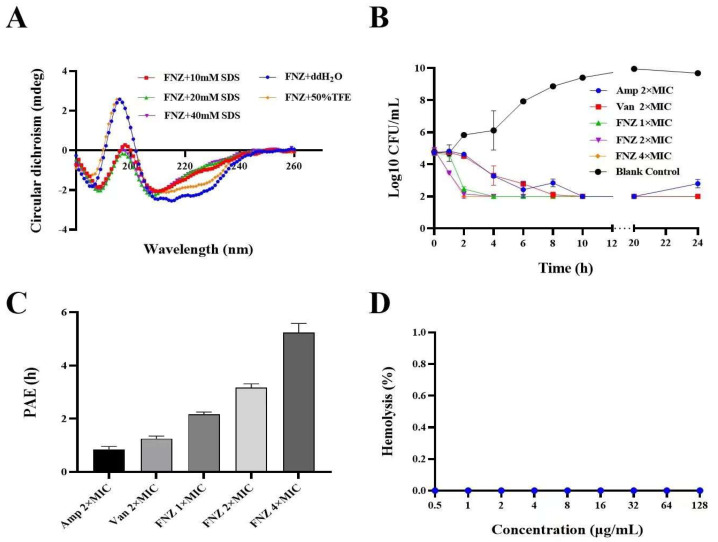
Antimicrobial characteristic of FNZ. (**A**) CD spectra of FNZ in water, SDS, and 50% TFE; (**B**) Time-killing curves of FNZ (1×, 2×, or 4× MIC) against *S. aureus* ATCC 43300; (**C**) The PAE assay of FNZ against *S. aureus* ATCC 43300. (**D**) Hemolytic activity of FNZ at different concentrations (0.5–128 μg/mL) against mouse erythrocytes. Amp: ampicillin, Van: vancomycin. The results are given as the mean ± SD (*n* = 3).

The ddH_2_O, sodium dodecyl sulfate (SDS), and 50% trifluoroethanol (TFE) were used to simulate water, bacterial, and eukaryotic cell membrane, and their secondary structures of peptide were determined [37]. In ddH_2_O and 50%TFE solutions, FNZ had a positive peak at 195 nm and two negative peaks at 206 nm and 230 nm, respectively. In SDS solutions of 10, 20, and 40 mM, FNZ has a positive peak at 200 nm and a negative peak at about 215 nm, indicating that the β-sheet is the main structure of FNZ in SDS solution (Figure 6A). 

### 2.7. Antimicrobial Activity Assays of FNZ

#### 2.7.1. Minimal Inhibitory Concentrations (MICs) of FNZ

It is essential to evaluate the antimicrobial activity of FNZ, because the first amino acid in the N-terminal of parental peptide was changed to Phe (F). As shown in Table 1, the FNZ had high antimicrobial activity to G^+^ bacteria (*S. aureus*, *S. hyicus*, *S. agalactiae*, *S. epidermidis*) with the MICs of 4–8 μg/mL. Meanwhile, it showed no activity to Gram-negative bacteria (*E. coli*, *S. enterica*, *P. aeruginosa*) with the MICs > 128 μg/mL.

#### 2.7.2. Synergism Assays of FNZ with Traditional Antibiotics

As shown in Table 2, the nisin, ampicillin, kanamycin, and vancomycin showed synergistic effect with FNZ, and the fractional inhibitory concentration index (FICI) for each were 0.325, 0.078, 0.07, and 0.25, respectively. Meanwhile, the combination of FNZ with ciprofloxacin showed antagonism as 1.531 of FICI. It was exciting that the MIC values reduced by 4–128 times in all synergistic combinations between FNZ and antibiotics to *S*. *aureus* ATCC 43300 (Table 2), showing a broad selective spectrum with other antimicrobials in future clinical trials and practices.

#### 2.7.3. Bactericidal Kinetics Assay

After being subjected to 1×, 2×, and 4× MIC of FNZ, the bacterial counts (log_10_ CFU/mL) of *S. aureus* ATCC 43300 decreased by 2.227, 2.752, and 2.894 within 2 h, respectively, all was more than 99% of decreased percentage. By contrast, there was a decrease of 0.101 and 0.216 log_10_ CFU/mL, respectively, after treatment with control antibiotics of ampicillin and vancomycin (2× MIC). Additionally, there was no growth of bacteria after 4 h of treatment with all concentrations of FNZ, showing superior to control antibiotics (Figure 6B). Furthermore, the number of colonies did not rebound until 24 h after treatment with FNZ, whereas the rebound situation was observed in the 2× MIC of ampicillin treatment.

#### 2.7.4. The Post−Antibiotic Effect (PAE) of FNZ against *S. aureus*

As shown in Figure 6C, the PAE of FNZ to *S. aureus* ATCC 43300 was prolonged (2.18, 3.13, and 5.27 h, respectively) with the increasing concentration (at 1× MIC, 2× MIC, and 4× MIC), whereas the PAE of vancomycin at 2× MIC was 1.3 h. The long PAE of FNZ makes it possible to extend the interval of medication and decrease the daily dosages.

### 2.8. Hemolysis, Cytotoxicity, and Stability of FNZ

#### 2.8.1. Hemolytic Assay and Cytotoxicity

**Figure 7:**
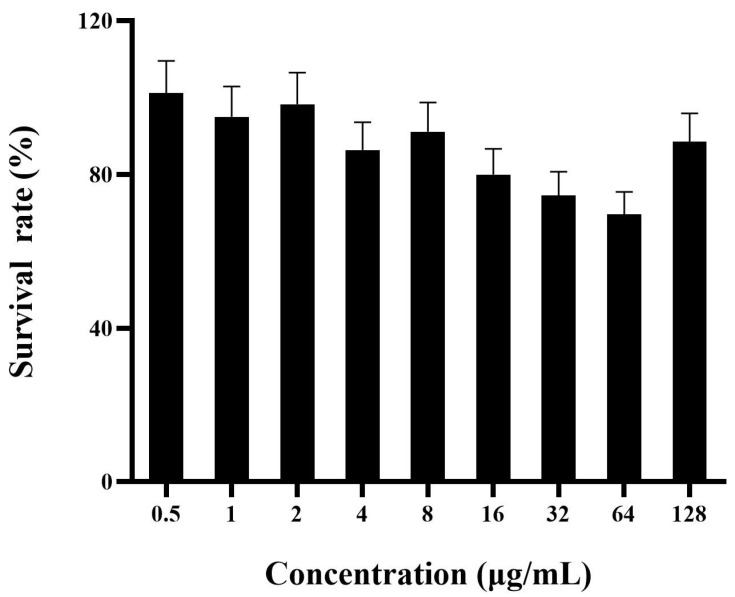
Cytotoxicity of FNZ against RAW264.7 cells. The results are given as the mean ± SD (*n* = 3).

Various concentrations of FNZ (from 0 to 128 μg/mL) were tested in hemolysis assay (Figure 6D). None of the concentrations of FNZ produced hemolytic activity on erythrocytes. The result demonstrated that there was no damage to mouse red blood cells (MRBCs) with FNZ in all scope-effective concentrations. Additionally, as shown in Figure 7, FNZ showed low cytotoxicity (88.56% survival) toward mouse macrophages RAW264.7 cells at 128 μg/mL, which suggested that FNZ might be a safe candidate drug for clinical application.

#### 2.8.2. Thermal, pH, Salts, and Protease Stability of FNZ

After being subjected to different temperatures (20, 40, 60, and 80 °C) for 1 h, no influence on the antibacterial activity of FNZ against *S. aureus* ATCC 43300 was observed. Meanwhile, the MIC of FNZ increased twice at 100 °C. FNZ exhibited a stable activity in the pH range from 2.0 to 8.0 (Table 3). The activity of FNZ was not as good in alkaline environments (pH 10) as in neutral or acidic environments (pH 2–8). It also maintains stability in different concentrations of mononuclear and divalent salt ions, but the activity decreased (MIC > 16 μg/mL) in the environment of artificial intestinal fluid.

## 3. Discussion

In recent 30 years, *P. pastoris* has become one of the mostly used tools for heterologous protein production [37,38,39,40]. Kex2 is a Ca^2+^-dependent serine protease which can specifically recognize and cut the carboxy–terminal peptide bond of Lys/Arg–Arg [41]. Combined with the yeast ER sequestration screening (YESS) and next-generation sequencing, it is confirmed that the P1’ site of Kex2 plays a key role in determining the cleavage and secretion of heterologous proteins [42]. It was found that the Ans in the P1’ site of Kex2 could increase over fourfold of luciferase as much compared to the Cys in the P1’ site [35]. Substitution of Glu by Val/Ala at Kex2 cleavage site P1’ could increase by 3 times the production of extracellular colony−stimulating factor [43]. However, there were no studies on P1’ site optimization to improve the yields of AMPs so far as we know. We designed 20 sequences (Table S1) with a different residue at the P1’ site of each other (Figure 1). Meanwhile, there is also a Ste13 restriction site in the α-factor signal peptide sequence, cleaving between Glu and Ala. This factor existing in protease has a certain degree of non-specific cleavage, which may result in a miscleavage on the target protein [44]. Therefore, the gene of Ste13 was removed when the recombinant vector was designed. Through subsequent expression verification, the four transformants (G1, F39, R33, and W25) with higher expression from the 48-well plates, 250 mL, and 1 L shake flask level were selected to express in 5 L fermenter. The transformant F39 had the highest yield with a level of 4.81 g/L (ferment supernatant) (Figure 4D). In addition, the target peptide FNZ from F39 could be directly purified by one−step ion exchange column. Thus, it is expected that it should be more suitable for the industrial aim of most target proteins due to its high expression, cleavage and easy purification.

The pro−leader of heterologous protein is translocated across the ER membrane to the lumen in yeast. The pre−region is cleaved by signal peptidase, which can guide the translocation of newborn peptide chains into the endoplasmic reticulum (ER); and the Kex2 protease recognizes and cuts Lys–Arg recognition sites. The pro−region ensures proper transport of newborn peptides from ER lumen to the Golgi apparatus, and the Ste13 protease cleavages two duplicate Glu–Ala recognition sites. Finally, the mature protein is secreted out of the cytoplasm [45]. The structure of α−signal peptide was simulated as the amino acid at P1’ was changed (Figure 5). The *S. cerevisiae* α-MAT signal leader is composed of a pre-region with 19 amino acids and a pro-region of 66 amino acids [45,46]. The latter regulates receptor-dependent packaging that causes the concentration of secreted proteins in the COPII transport vesicles derived from the endoplasmic reticulum (ER) [31,47]. In this study, the secondary structure of the pre-peptide is still characteristic of multiple α-helices. However, β-sheet was dominant in the wild-type α-MAT pro-region, and three β-sheet structures were formed at amino acids 40–42, 51–54, and 63–65. In mutant α-MAT pro-region, α-helix was dominant, amino acids 34–38 and 68–75 form two α-helical structures, so the secondary structure of the mutant is highly different from that of the wild type, changing tendency from β-sheet to α-helix, which may allow the secreted protein to escape from the receptor and enter more easily into the Golgi apparatus from the vesicle, thus increasing the secretion level of the protein.

Due to the addition of the first amino acid in the N-terminal, it is essential to evaluate the antimicrobial characteristics of FNZ as a new peptide. We found that FNZ has narrow spectrum antibacterial activity similar to NZ2114 against target G^+^ bacteria [48,49] mainly to *S. aureus*, and *Streptococcus* sp. with the MICs ranging from 4 to 8 μg/mL (Table 1). However, the activity of FNZ reduced by twofold compared with parental peptide NZ2114 (Table 1). This might be attributed to new F at the P1’ site, being as a bigger hydrophobic side-chain structure from the benzene ring group. Yes, its exact structure and mechanism should be preferentially studied in further study. 

Although the activity of FNZ to some pathogens decreased, the other more merits of druggability were observed. The killing rate of FNZ against *S. aureus* ATCC 43300 within 2 h was more than 99% at 2× and 4× MIC, which was better than those of vancomycin and ampicillin (Figure 6B). Furthermore, the number of colonies did not rebound until 24 h after treatment with FNZ, whereas the parental peptide NZ2114 could not inhibit bacterial regrowth after 6 h of inoculation for *S. aureus* ATCC 43300 (Table 4). The long drug efficacy span might be helpful to reduce the frequency of drug usage and prevent the occurrence of drug resistance. The PAE reflects the inhibitory effect on the growth of pathogen after short contact with the drug, which is related to the bactericidal characteristic of the drug [50]. The PAEs of FNZ were 2.18, 3.13, and 5.27 h at 1× MIC, 2× MIC, and, 4× MIC, which are longer than that of NZ2114 at the same concentration (1.7, 2.6, and 3.5 h at 1× MIC, 2× MIC, and 4× MIC) (Table 4) and vancomycin at 2× MIC (Figure 6C). These results suggested that FNZ has the potential as a potent agent against *S. aureus,* especially MRSA strains. 

It has been shown that the combination of antimicrobial drugs is one of the strategies to improve antibacterial activity and retard drug resistance [51]. In this work, FNZ showed synergistic effect with antibiotics such as ampicillin, kanamycin, vancomycin, and nisin with the FICI < 0.5 (Table 2), which was similar to the parental peptide NZ2114 (Table 4). This synergistic effect could significantly reduce the dose of antibiotics, effectively alleviating the development of antibiotic resistance of pathogens [52]. 

Maximizing antibacterial activity of AMPs with minimal host toxicity is an attractive direction [53]. A hemolysis test is mainly used to detect the rupture of red blood cells and determine whether drugs are harmful in vivo [54]. The NZ2114 showed no hemolysis in human MRBCs, while it had high host toxicity (cell survival rate: 52.13% at 128 μg/mL) to RAW264.7 cells (Table 4). In this study, FNZ showed no hemolysis and lower cytotoxicity (cell survival rate: 88.56%) even at 128 μg/mL, which was consistent with the decrease of activity caused by the change of the P1’ site. The π–π stacking between the benzene rings on the Phe side chain of the peptide could cause the hydrophobic part to tend to gather internally, reducing the surface area in contact with the polar environment and side effects in vivo, especially at the high peptide concentration [55]. Nevertheless, the high biosafety assures the FNZ a higher safe level as candidate drug for clinical usage. Meanwhile, similar to NZ2114 (Table 4), FNZ could retain half activity even when exposed to 100 °C for 1 h (Table 3). The high cysteine content might contribute to its thermal stability. AMPs act with the bacterial membrane through an interaction between a positively charged peptide and a negatively charged bacterial membrane [56,57,58]. This interaction subsequently leads to inactivation of the bacteria. However, the increasing concentration of cations in the environment can prevent the peptide from interacting with the membrane, thus nulling the efficiency to kill bacteria. In this work, FNZ showed strong antimicrobial activity against *S. aureus* ATCC 43300 in the conditions of 300 mM Na^+^ solution and 150 mM Mg^2+^ solution (Table 3). This result indicates that FNZ is insensitive to the presence of cations and remains the antimicrobial activity in the presence of high concentrations of monovalent or divalent cations. Generally, the above main characteristics showed that FNZ meets the key needs as the new candidate of antimicrobial drug in treatment of G^+^ pathogenic bacteria in the aspect of safety, bioactivity, and stability as a whole.

## 4. Materials and Methods

### 4.1. Plasmids, Strains, and Reagents

The plasmid pPICZαA and *P. pastoris* X−33 strain were purchased from Invitrogen. *Salmonella enterica* ATCC 13076, *Staphylococcus epidermidis* ATCC 12228, *Escherichia coli* ATCC 25922, *S. aureus* ATCC 43300, and *S. aureus* ATCC 25923 were obtained from the American Type Culture Collection, *S. aureus* CVCC 546 was purchased from the China Veterinary Culture Collection Center. *Pseudomonas aeruginosa* CICC 20625 was purchased from the China Center of Industrial Culture Collection, *Streptococcus agalactiae* CAU−FRI 4 was offered by the College of Veterinary Med China Agricultural University, *S. aureus* E48 by the College of Animal Sci and Technol Northwest A&F University, and clinical *Staphylococcus hyicus* ACCC 61734 by the Tianjin Institute of Animal Husbandry and Veterinary Med Tianjin Academy of Agricultural Sciences. All reagents were analytical grade.

### 4.2. Construction of the Expression Vector pPICZαA-X-NZ2114

A 20 DNA sequences of NZ2114 derivatives, with one different amino acid at the P1’ site of each other, were synthesized by Sangon Biotech (Shanghai, China) Co., Ltd. and inserted into pPICZαA vector, generating 20 recombinant vectors pPICZαA-X_(1–20)_-NZ2114. After linearizing by *Pme*I and transforming into *P. pastoris* X-33 by electroporation [18], positive transformants were selected on YPDS plates with 100 μg/mL zeocin.

### 4.3. Expression of the XNZ in P. pastoris in 48-Well Plates and Shake Flasks

Transformants were inoculated into 48-well plates containing 500 μL of BMGY medium. A 0.5% final concentration of methanol as inducer was added every 24 h. The supernatants were collected and conducted by inhibition zone assay [18]. A single colony with strong activity was cultured in shake flasks containing 50 mL and 250 mL BMGY medium, for first and re-screening respectively. The expression product was analyzed by tricine–dodecyl sodium sulfate–polyacrylamide gel electrophoresis (Tricine–SDS–PAGE) [22]. 

### 4.4. High-Density Cultivation and Purification

The 5 L level fermentation and purification were performed according to previous protocols [18,59].

### 4.5. Structure Modeling of Wild-Type and Mutant Pro-Signal Peptides

According to the amino acid sequence of the α-MAT signal peptide (NCBI AGW24899.1), the 3D structures of original and P1’-modified α-MAT signal peptides were performed by I-TASSER [60]. Then, the difference between the two signal peptides structure was compared by the PyMol 2.3.0. The amino acid sequences of α-MAT pro-signal peptide for XNZ mutant are shown in the Supplementary Materials.

### 4.6. Secondary Structure Determination of FNZ

The secondary structures of FNZ in ddH_2_O, SDS, and 50% TFE environments were analyzed by Circular Dichroism spectrum (CD) at room temperature. The volume of 200 μL peptide solution was added into a 1 mm path-length cuvette [61]. The spectra were recorded from 185 to 260 nm, and the scanning speed was 10 nm/min. Each sample was scanned 3 consecutive times, and the average value was calculated. 

### 4.7. Antimicrobial Characteristics of FNZ

#### 4.7.1. Minimal Inhibitory Concentration (MIC)

The minimal inhibitory concentrations (MICs) assay was performed according to the CLSI procedures with the final peptide concentrations of 256, 128, 64, 32, 16, 8, 4, 2, 1, and 0.5 μg/mL, respectively. All tests were conducted in triplicate [62].

#### 4.7.2. In Vitro Pharmacodynamics of FNZ

The *S. aureus* ATCC 43300 in logarithmic growth stage was diluted to a final concentration of 10^6^ CFU/mL. The peptide solutions were mixed with bacteria suspension in the ratio of 1:9, and the final concentrations of FNZ were 1×, 2×, and 4× MIC, respectively. Samples were taken at 0, 0.5, 1, 2, 4, 6, 8, 10, 20, and 24 h, serially diluted, and visible colonies were counted on MHA plates [63]. The positive controls were vancomycin and ampicillin.

#### 4.7.3. The PAE of FNZ 

The 10^8^ CFU/mL *S. aureus* ATCC 43300 in mid-log phase was incubated to 1×, 2×, and 4× MIC of FNZ for 2 h. The PBS was used as a blank control, and vancomycin with the concentration of 2× MIC was used as a positive control. After the exposed time, the bacteria suspension was reconstructed by dilution to 1000 times with MHB medium to remove the influence of peptides and antibiotics. Samples were taken every hour, and then coated on MHA plate after gradient dilution. The calculation formula was from previous studies [64]. 

#### 4.7.4. Drug Synergism Assays of FNZ with Traditional Antibiotics 

The checkerboard method was used to determine the interaction between FNZ and other antimicrobial drugs, which were vancomycin, ampicillin, ciprofloxacin, kanamycin, and nisin. The diluted samples with the concentrations of 1/16 to 8× MIC were added into the 96-well plates and incubated at 37 °C for 16 h. The effects of combination were evaluated by calculating the FICI. FICI ≤ 0.5 was defined as synergy, 0.5 < FICI ≤ 1 was defined as additivity, 1 < FICI ≤ 4 was defined as indifference, and FICI > 4 was defined as antagonism [65,66].

### 4.8. Hemolysis, Cytotoxicity, and Stability of FNZ

#### 4.8.1. Hemolysis

The FNZ with the concentrations of 256, 128, 64, 32, 16, 8, 4, 2, and 1 μg/mL was mixed with the 8% MRBCs suspension (diluted with 0.9% NaCl) in the ratio of 1:1. Then, the supernatants were collected by centrifugation at 1844× *g* for 5 min at 4 °C, and the absorption value was measured at 540 nm. Hemolysis (%) = [(A − A_0_)/(A_100_ − A_0_)] × 100%, A_0_: the blank control of 0.9% NaCl; A_100_: the positive control of 0.1% Triton X-100 [67].

#### 4.8.2. Cytotoxicity

The MTT assay was used to test the cytotoxicity of FNZ to the RAW 264.7 cells [68]. 

#### 4.8.3. Stability 

The tolerance of FNZ to temperature and pH was evaluated [69]. The stability of FNZ in simulated gastric fluid, artificial intestinal fluid and mouse serum was carried out as previously described [70]. Sodium ions (Na^+^) and magnesium ions (Mg^2+^) at concentrations of 50, 100, 150, 300, and 500 mM were incubated with peptide solution at 37 °C for 1 h, and their MIC values were measured [71]. All assays were conducted in triplicate.

## 5. Conclusions

In summary, the P1’ site of Kex2 was optimized by replacing the P1’ site with 20 amino acids, in turn, to enhance the expression level of NZ2114. It was shown that the yield significantly increased from the original 2.39 g/L to 4.81 g/L when the amino acid of the P1’ site changed to F. Additionally, the novel candidate peptide FNZ also showed a strong antibacterial activity against G^+^ bacteria, especially to *S. aureus* and *S. agalactiae* (MIC: 4–8 μg/mL) despite it decreased by twofold compared with the parental peptide (Table 1). The FNZ was quite stable and retained high activity in various conditions. There was no hemolysis and low cytotoxicity even at a high concentration of 128 μg/mL. Above results show a successful harvest for molecular modification on Kex2 P1’ site with the positive responses including expression, cleavage, bioactivities as antimicrobial characteristics and other druaggabilities. Thus, this updated yeast expression system might be tried to extend into the heterogeneous expression of other similar peptides and proteins at industrial scale depending on more trials case by case in the future.

## Figures and Tables

**Table 1:** Antibacterial spectrum of the FNZ.

Strains	MIC							
	FNZ		NZ2114		Ampicillin		Vancomycin	
	μg/mL	μM	μg/mL	μM	μg/mL	μM	μg/mL	μM
**Gram-positive bacteria**								
*Staphylococcus aureus* ATCC 43300	8	1.75	4	0.91	2	2.48	1	0.67
*S. aureus* ATCC 25923	8	1.75	4	0.91	0.25	0.62	4	2.69
*S. aureus* CVCC 546	8	1.75	8	1.81	0.25	0.62	1	0.67
*S. aureus* E48	4	0.88	NT	NT	0.25	0.62	1	0.67
*S. hyicus* ACCC 61734	8	1.75	4	0.91	NT	NT	NT	NT
*S. epidermidis* ATCC 12228	8	1.75	NT	NT	2	2.48	1	0.67
*S. agalactiae* CAU-FRI 4	4	0.88	2	0.45	NT	NT	NT	NT
**Gram-negative bacteria**								
*Escherichia coli* ATCC 25922	>128	>28.05	>128	>28.97	1	1.24	128	86
*Salmonella enterica* ATCC 13076	>128	>28.05	>128	>28.97	NT	NT	>128	>86
*Pseudomonas aeruginosa* CICC 20625	>128	>28.05	>128	>28.97	NT	NT	>128	>86

NT: no test.

**Table 2:** Combination effects of FNZ with antibiotics against *S*. *aureus* ATCC 43300.

Combination	Variety	*S. aureus* ATCC 43300			
		MICa (μg/mL)	MICc (μg/mL)	FIC	FICI
FNZ + Amp	FNZ	8	0.125	0.016	0.078
	Amp	2	0.125	0.063	
FNZ + Kan	FNZ	8	0.5	0.063	0.07
	Kan	64	0.5	0.008	
FNZ + Nisin	FNZ	8	0.5	0.078	0.328
	Nisin	4	1	0.25	
FNZ + Cip	FNZ	8	0.25	0.031	1.531
	Cip	0.25	0.375	1.5	
FNZ + Van	FNZ	8	1	0.125	0.25
	Van	1	0.125	0.125	

Amp: ampicillin, Kan: kanamycin, Van: vancomycin, Cip: ciprofloxacin, MICa: the MIC of drug alone, MICc: the MIC of drug used in combination.

**Table 3:** Thermal, pH, salts, and protease stability of FNZ against *S. aureus* ATCC 43300.

Treatment	MIC			
	FNZ		Vancomycin	
	μg/mL	μM	μg/mL	μM
**Temperature (°C)**				
20	8	1.75	1	0.67
40	8	1.75	1	0.67
60	8	1.75	1	0.67
80	8	1.75	1	0.67
100	16	3.5	1	0.67
**pH**				
2	8	1.75	1	0.67
4	8	1.75	1	0.67
6	8	1.75	1	0.67
8	8	1.75	1	0.67
10	16	3.5	1	0.67
**Salts**				
50 mM Na^+^	8	1.75	1	0.67
100 mM Na^+^	8	1.75	1	0.67
150 mM Na^+^	8	1.75	1	0.67
300 mM Na^+^	8	1.75	1	0.67
500 mM Na^+^	16	3.5	1	0.67
50 mM Mg^2+^	8	1.75	1	0.67
100 mM Mg^2+^	8	1.75	1	0.67
150 mM Mg^2+^	8	1.75	1	0.67
300 mM Mg^2+^	16	3.5	1	0.67
500 mM Mg^2+^	>32	>7	1	0.67
**Simulated Gastric Fluid** (1 h)	8	1.75	1	0.67
**Artificial Intestinal Fluid** (10 min)	>16	>3.5	1	0.67
**Serum** (25%) (1–4 h)	8	1.75	1	0.67

**Table 4:** Expression level and druggability of FNZ and NZ2114.

Index	FNZ	NZ2114
**Expression level**		
Yield (g/L)	4.81 ^a^	2.31 ^b^
Specific production rates (mg/g/h)	0.095 ^a^	0.038 ^b^
**Druggability**		
MICs (μg/mL)	*S. aureus*: 4–8 ^a^ *S. hyicus*: 8 ^a^ *S. agalactiae*: 4 ^a^	*S. aureus*: 4–8 ^a^ *S. hyicus*: 4 ^a^ *S. agalactiae*: 2 ^a^
Bactericidal kinetics	>99% *S. aureus* were killed ^a^ No regrowth within 24 h ^a^	>99% *S. aureus* were killed ^b^ Bacterial regrowth was observed after 6 h of inoculation ^b^
PAE (h)	2.18, 3.13, and 5.27 h at 1× MIC, 2× MIC, and 4× MIC ^a^	1.7, 2.6, and 3.5 h at 1× MIC, 2× MIC, and 4× MIC ^b^
Synergism	Synergistic effect with ampicillin, kanamycin, vancomycin, and nisin ^a^	Synergistic effect with ampicillin, kanamycin, vancomycin, and streptomycin ^b^
Hemolysis (%)	0% ^a^	Less than 0.1% ^b^
Cell viability (%)	88.56% ^a^ at 128 μg/mL	52.13% ^c^ at 128 μg/mL
Thermal stability	Stable at 20 to 80 °C ^a^ 50% activity at 100 °C ^a^	Stable at 20 to 80 °C ^b^ 20% activity at 100 °C
pH stability	Stable at pH 2–8 ^a^ 50% activity at pH 10 ^a^	High activity at pH 8 and 10 ^b^ Low activity at pH 2 to 6 ^b^
Salts stability	Stable at 50–300 mM Na^+ a^ 50% activity at 500 mM Na^+ a^ Stable at 50–150 mM Mg^2+ a^ 50% activity at 300 mM Mg^2+ a^ Lower than 50% activity at 500 mM Mg^2+ a^	NT
Serum stability	Stable^a^	NT

^a^: data from this study; ^b^: data from ref 18; ^c^: data from ref 68; NT: no test.

## Data Availability

The original contributions presented in the study are included in the article; further inquiries can be directed to the corresponding author(s).

## Supplementary Materials

The following supporting information can be downloaded at: https://www.mdpi.com/article/10.3390/antibiotics12040786/s1, Table S1: The amino acid sequences of 20 new constructs; Figure S1: The antibacterial activity of FNZ purified collected peak was identified by inhibition zone assay.
