# Supplementary material for: Molecular Modification of Kex2 P1’ Site Enhances Expression and Druggability of Fungal Defensin
Source: Antibiotics (Basel). 2023 Apr 20;12(4):786. doi: 10.3390/antibiotics12040786 (PMC10135057; doi:10.3390/antibiotics12040786)
Supplement: Supplementary file 1 [file antibiotics-12-00786-s001.zip › antibiotics-2313888-supplementary.pdf]

Supplementary materials

Molecular Modification of Kex2 P1' Site Enhances Expression and Druggability of Fungal Defensin

Yanjie Jin [1,2,3,†], Na Yang [1,2,3,†], Da Teng [1,2,3], Ya Hao [1,2,3], Ruoyu Mao [1,2,3,*] and Jianhua Wang [1,2,3,*]

1 Gene Engineering Laboratory, Feed Research Institute, Chinese Academy of Agricultural Sciences, Beijing 100081, China
2 Innovative Team of Antimicrobial Peptides and Alternatives to Antibiotics, Feed Research Institute, Chinese Academy of Agricultural Sciences, Beijing 100081, China
3 Key Laboratory of Feed Biotechnology, Ministry of Agriculture and Rural Affairs, Beijing 100081, China
* Correspondence: maoruoyu@caas.cn (R.M.); wangjianhua@caas.cn (J.W.)
† These authors contributed equally to this work.

Table S1. The amino acid sequences of 20 new constructs.

New constructs (XNZ)	Amino acid sequence
NZ2114	N'- GFGCNGPWNEDDLRCHNHCKSIKGYKGGYCAKGGFVCKCY (40)
ANZ	A--CY
CNZ	C--CY
DNZ	D--CY
ENZ	E--CY
FNZ	F--CY
GNZ	G--CY
HNZ	H--CY
INZ	I--CY
KNZ	K--CY
LNZ	L--CY
MNZ	M--CY
NNZ	N--CY
PNZ	P--CY
QNZ	Q--CY
RNZ	R--CY
SNZ	S--CY
TNZ	T--CY
VNZ	V--CY
WNZ	W--CY
YNZ	Y--CY

NT: The dotted line indicates the same amino acid sequence as NZ2114 except 1st residue.

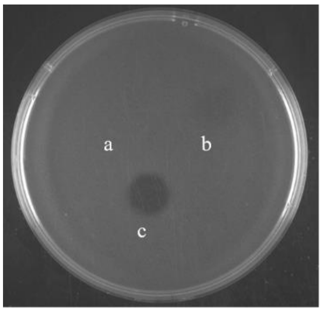

Figure S1. The antibacterial activity of FNZ purified collected peak was identified by inhibition zone assay. a: penetration peak; b: 27.5% B eluting peak; c: 60% B eluting peak, 20 μL of every collected peak. The tested strain was *S. aureus* ATCC 43300.
